# Molecular alterations and prognosis of breast cancer with cutaneous metastasis

**DOI:** 10.1186/s13000-024-01509-x

**Published:** 2024-07-05

**Authors:** Yan Xu, Li Ding, Chao Li, Bin Hua, Sha Wang, Junli Zhang, Cuicui Liu, Rongyun Guo, YongQiang Zhang

**Affiliations:** 1 Department of Oncology, Beijing Hospital, National Center of Gerontology, Institute of Geriatric Medicine, Chinese Academy of Medical Sciences, Beijing, People’s Republic of China; 2 Geneseeq Research Institute, Geneseeq Technology Inc, Nanjing, Jiangsu China

**Keywords:** Cutaneous metastasis, Breast cancer, Next-generation sequencing-NGS, Genomic analysis, Prognosis

## Abstract

**Purpose:**

Cutaneous metastasis (CM) accounts for 5–30% of patients with breast cancer (BC) and presents unfavorable response to treatment and poor prognosis. A better understanding of the molecular alterations involved in metastasis is essential, which would help identify diagnostic and efficacy biomarkers for CM.

**Materials:**

: We retrospectively reviewed a total of 13 patients with histological or cytological diagnosis of breast cancer and CM. Clinical information was extracted from the medical records. The mutational landscape of matched primary tumors with their lymph nodes or CM tissues were analyzed using next-generation sequencing (NGS) of 425 cancer-relevant genes. All tissues were also analyzed by immunohistochemistry (IHC). The association of prognosis with various clinical and molecular factors was also evaluated.

**Results:**

More than half of the patients were Ki67 low (< 50%, 53.7%). Most patients (12, 92.3%) had other metastasis sites other than skin. The median time from diagnosis to the presentation of CM (T1) was 15 months (range: 0–94 months) and the median time from CM to death (T2) was 13 months (range 1–78). The most frequently altered genes across the three types of tissues were *TP53* (69.6%, 16/23), *PIK3CA* (34.8%, 8/23), and *MYC* (26.1%). The number of alterations in CM tends to be higher than in primary tumors (median 8 vs. 6, *P* = 0.077). Copy number loss in *STK11*, copy number gain in *FGFR4, TERT, AR, FLT4* and *VEGFA* and mutations in *ATRX, SRC, AMER1* and *RAD51C* were significantly enriched in CM (all *P* < 0.05). Ki67 high group (> 50%) showed significantly shorter T1 than the Ki67 low group (≤ 50%) (median 12.5 vs. 50.0 months, *P* = 0.036). *TP53*, *PIK3CA* mutations, and *TERT* amplification group were associated with inferior T2 (median 11 vs. 36 months, *P* = 0.065; 8 vs. 36 months, *P* = 0.013, 7 vs. 36 months, *P* = 0.003, respectively). All p values were not adjusted.

**Conclusion:**

We compared the genomic features of primary breast cancer tissues with their corresponding CM tissues and discussed potential genes and pathways that may contribute to the skin metastasis of advanced breast cancers patients. *TP53*, *PIK3CA* mutant, and *TERT* amplification may serve as biomarkers for poor prognosis for CM patients.

**Supplementary Information:**

The online version contains supplementary material available at 10.1186/s13000-024-01509-x.

## Introduction

Breast cancer (BC) stands as the most prevalent malignancy among females worldwide, with cutaneous metastasis (CM) occurring in 5–30% of BC patients [1]. CM not only impacts patients’quality of life but also correlates with unfavorable treatment responses and poor prognosis [2, 3]. Developing innovative therapeutic strategies for CM poses a significant clinical challenge, given the poor prognostic outcomes and the complex molecular alterations underlying metastasis. Unfortunately, there is a scarcity of studies on the genomic profiling of breast cancer with CM, and the findings thus far are inconclusive. Some studies indicated that gene alterations in matched primary tumors and distant metastases (including skin) share highly similarity. Another study involving 33 breast cancer patients revealed that triple-negative type has a higher risk of metastasis to the skin, with nearly half of the CM cases exhibiting additional molecular alterations compared to their corresponding primary tumors. However, due to considerable interpatient variability, no distinct patterns associated with cutaneous metastatic development have been observed [4].

In this study, we conducted a retrospectively analyzed 13 patients with CM from breast cancer. Through next-generation sequencing (NGS), we profiled the mutational features in both primary tumors and CM, thereby identifying potential molecular associations with CM and its prognosis.

## Materials and methods

### Patients and samples

We retrospectively reviewed a total of 13 patients with histological or cytological diagnosis of breast cancer and CM in Beijing Hospital from January 2018 to December 2020. Those cutaneous metastases were not from the skin over the breast. This study was approved by the Ethical Committee of Beijing Hospital (Approval No. 2020BJYYEC-062-05). The patients/participants provided their written informed consent to participate in this study. Of those 13 patients, 8 had matched primary tumors and CM, 1 had primary tumor only, and 4 had CM tumor sample only. Of the patients with paired samples, 2 patients had lymph nodes samples. Clinical information, including age at diagnosis, disease stage, metastasis sites, and treatment history were extracted from the medical records. T1 was defined as the time between diagnosis and the presentation of CM, and it was also defined as the disease free interval (DFI) of CM; T2 was defined as the time from the presentation of CM to death, and it was also defined as the overall survival (OS). Targeted sequencing with a 425 cancer-related gene panel was performed with the primary and/or CM tumor tissue sample from each patient (gene list, Table S1). The mutation list of tumor samples is shown in Table S2.

### Immunohistochemistry

All 9 primary tumors and 12 metastases underwent immunohistochemical study for the expression of estrogen receptors (ER), progesterone receptors (PR), HER2 and Ki67. Immunostaining was performed using the EnVision detection system (UltraPATH 30, Zhongshan Golden Bridge Biotechnology Co. Ltd, Beijing, China) and the following antibodies: ER (clone EP1, Zhongshan Golden Bridge Biotechnology Co. Ltd, Beijing, China), PR (clone EP2, Zhongshan Golden Bridge Biotechnology Co. Ltd, Beijing, China), and Ki-67 (clone UMAB107, Zhongshan Golden Bridge Biotechnology Co. Ltd, Beijing, China). The HER2 immunostaining was performed using the EnVision detection system (Benchmark ultra, F. Hoffmann-La Roche Ltd, Switzerland) and the antibody (clone 4B5, F. Hoffmann-La Roche Ltd. Switzerland). Evaluation of ER, PR, and HER2 expression was performed according to American Society of Clinical Oncology and the College of American Pathologists (ASCO-CAP) guidelines. HER2 equivocal cases (2+) underwent FISH analysis, using the HER/CEP17-2DNAProbe Kit (Wuhan HealthCare Biotechnology Co., Ltd. Hubei, China) on complete tumor sections. Results were interpreted according to 2018 ASCO-CAP guidelines [5].

### DNA extraction and sequencing library preparation

The genomic DNA from formalin-fixed and paraffin‐embedded (FFPE) was extracted using the QIAamp DNA FFPE Tissue Kit (Qiagen) according to the manufacturer’s protocol [6]. The quantity and quality of the extracted DNA were evaluated using a Qubit 3.0 fluorometer and Nanodrop 2000, respectively (Thermo Fisher Scientific). Sequencing libraries were prepared using the KAPA Hyper Prep Kit (KAPA Biosystems) according to the manufacturer’s suggestions for different sample types. In brief, 1 µg of fragmented genomic DNA underwent end-repairing, A-tailing, and ligation with indexed adapters sequentially, followed by size selection using Agencourt AMPure XP beads (Beckman Coulter). Hybridization-based target enrichment was carried out with a pan-cancer gene panel (425 cancer-relevant genes), and xGen Lockdown Hybridization and Wash Reagents Kit (Integrated DNA Technologies). Captured libraries by Dynabeads M-270 (Life Technologies) were amplified in KAPA HiFi HotStart ReadyMix (KAPA Biosystems) and quantified by qPCR using the KAPA Library Quantification Kit (KAPA Biosystems) for sequencing.

### Targeted NGS and sequencing data Processing

Sequencing data were processed as previously described [6]. In brief, the data was first demultiplexed and subjected to FASTQ file quality control to remove low-quality data or N bases. Qualified reads were mapped to the reference human genome hg19 using Burrows-Wheller Aligner and Genome Analysis Toolkit (GATK 3.4.0) was employed to apply the local realignment around indels and base quality score recalibration. Picard was used to remove PCR duplicates. VarScan2 was employed for the detection of single-nucleotide variations (SNVs) and insertion/deletion mutations. SNVs were filtered out if the mutant allele frequency (MAF) was less than 1% for tumor tissue and 0.3% for plasma samples. Common SNVs were excluded if they were present in > 1% population in the 1000 Genomes Project or the Exome Aggregation Consortium (ExAC) 65,000 exomes database. The resulting mutation list was further filtered by an in-house list of recurrent artifacts based on a normal pool of whole blood samples. Parallel sequencing of matched white blood cells from each patient was performed to further remove sequencing artifacts, germline variants, and clonal hematopoiesis. The Copy number alterations were analyzed as previously described [7, 8]. The tumor purities were first estimated using ABSOLUTE [9]. Somatic CN alteration events were assigned based on sample-ploidy values calculated in the FACETS algorithm. Structural variants were detected using FACTERA with default parameters [10]. The fusion reads were further manually reviewed and confirmed on Integrative Genomics Viewer (IGV).

### Analysis of the mechanisms of CM

Gene list involving metastasis according to the previous studies [11–14], including *STK11*, *MYC*, *FGFR1/4*, *CDK12*, *TERT*, *AR*, *CBLB*, *BAK1*, *FLT1, ATRX, CREBBP*, *CHD8*, *PDK1, ALK, EZH2, MRE11A, SRC, ADGRB3, GATA3, XPA, PLCB4, DPYD, PTCH1, AXIN2, MET, ERBB4, FOXA1, NOTCH1, AMER1, ARID1A, ATR, EGFR, NSD1, RAD51C* and *FOXP1* was used to evaluate the status of alteration in all samples. The results derived from our patient cohort were further validated using a published independent dataset consisting of 807 patients with breast cancer [15]. Detailed clinicopathological features of the validation cohort can be found in Table S3.

### Statistical analysis

The concordance of genomic alterations between primary breast tumors and CMs was assessed using Fisher’s exact test. The Kaplan-Meier method was used for survival analyses, and statistical significance was assessed using the log-rank test. Hazard ratios (HRs) and 95% confidence intervals (CIs) were calculated from the Cox regression model. A two-tailed P-value < 0.05 was considered statistically significant. All statistical analyses were performed using R version 3.4.2.

## Results

### Overview of patient cohort

The clinicopathological characteristics of the 13 patients were summarized in Table 1. The median age at diagnosis was 50 years old (range: 30–70). Based on the results of immunohistochemical tests, 4 patients (48.5%) were Luminal HER2-, 3 patients (9%) were Luminal HER2+, 1 patient (6%) was HER2+ (non-Luminal), and 5 cases (36.4%) were TNBC. Five patients were PR + and ER+. Among the 13 patients, 11 had a high level of Ki67 expression (> 20%),and the remaining two patients had a Ki67 expression of 20%. The median Ki67 expression was 50%. Thus, we adjusted the cutoff value to 50% to better stratify Ki67 expression into low and high categories, resulting in 7 patients classified as Ki67 low (< 50%, 53.7%). The majority of patients (12, 92.3%) exhibited metastasis at sites other than the skin, with 7 of them having more than two additional metastasis sites. The most common metastatic sites were bone (58.3%, 7/12) and lymph nodes (41.6%, 5/12).

**Table 1:** Overview of patients’ clinicopathological characteristics

Characteristics	*N*, % of patients
Sex	
Male	0 (0.0%)
Female	13 (100%)
Age	
Median age (range)	50 (30 ~ 70)
Immunohistochemical markers	
ER+	7 (53.8%)
PR+	6 (46.3%)
ER + and PR+	5 (38.5%)
HER2+	4 (30.8%)
Ki67	
<50	7 (53.9%)
≥50	5 (38.5%)
Unknow	1 (7.7%)
Median (range)	48 (20 ~ 70)
Molecular type	
Luminal A^a^	0(0.00%)
Luminal B	7 (53.8%)
HER2 enriched	1 (7.7%)
TNBC	5 (38.5%)
Other metastasis sites	12 (92.3%)
lymph node	5 (38.5%)
bone	7 (53.9%)
liver	4 (30.8%)
lung	3 (23.1%)
Others^b^	3 (23.1%)
more than two sites	7 (53.9%)
Time from diagosis of BC to CM(T1)	
Median time (month, range)	15 (0 ~ 94)
Survival time after CM(T2)	
Median time (month, range)	13 (1 ~ 78)

^a^ the Ki67 less than 20% has been defined as luminal A

^b^ include spleen, chest wall and pleura

### The potential mutational correlation between primary tumors and metastases

**Fig. 1:**
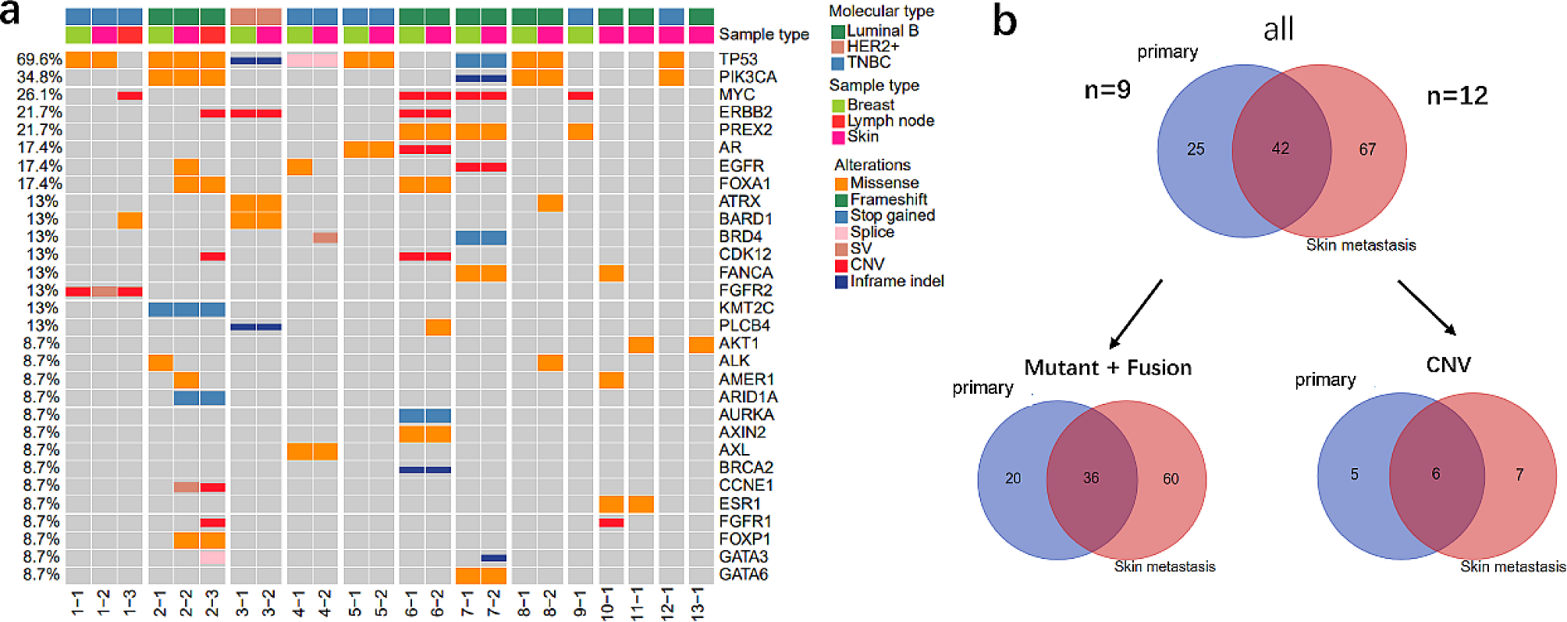
Mutation profiling of the primary and metastasis tumors. (**a**) Genomic landscape of the 13 patients. Molecular type and sample type were indicated by the bar on the top; the patient ID were indicated at the bottom. The types of alterations were indicated by different colors. Each column represented one sample of one patient. (**b**) Shared and specific mutations in 9 primary tumor and the 12 cm. The top pie chart showed all the variation types, including site mutation, fusion, and copy number variation (CNV); the two charts below displayed the shared and specific site mutation plus fusion, and CNV, separately

Nine primary tumors, 12 cm and 2 lymph nodes were analyzed using NGS with a 425 cancer-associated-gene panel. As shown in Fig. 1a, the most frequently altered genes across the three types of tissues were *TP53* (69.6%, 16/23), *PIK3CA* (34.8%, 8/23), *MYC* (26.1%), *ERBB2* (21.7%, 6/23), *PREX2* (21.7%, 5/23) and *AR* (17.4%, 4/23). Two patients had paired breast, CM and lymph node metastasis samples: patient 1 exhibited one shared mutation in *TP53* between the primary tumor and CM, one shared copy number amplification in *FGFR2* between the primary tumor and lymph node, no shared alterations between CM and lymph node, and two specific alterations in the lymph node. Meanwhile, patients 2 showed 6 shared alterations between CM and lymph node, 3 between the primary tumor and CM, and 2 between the primary tumor and lymph node (Fig. < link rid="fig1”>1</link>a, < link rid="fig1”>1</link>–1,2,3, 2 − 1,2,3).

Specifically, we compared the alteration concordance and divergence in 9 primary tumor and the 12 cm (Fig. 1b). Overall, 25 and 67 unique alterations (mutation, fusion and CNV) were detected in primary tumors and CM, respectively; 42 alterations were shared between the two. Considering the types of gene alteration, 20 mutations and gene fusions were specific to primary tumors, whereas 60 were specific to CM, with 36 shared alterations. Additionally, 5 CNVs were specific to primary tumors and 7 to CM, with 6 shared between the two. In the 8 patients who had matched primary tumor and CM samples, the number of alterations in CM tended to be higher in CMs than in primary tumors (median 8 vs. 6, *P* = 0.077) (Fig. S1a), suggesting the presence of more sub-clonal variations in CMs. Moreover, the proportion of CM-specific alterations varied greatly among patients, ranging from 8.33 to 66.7% (Fig. S1b).

### Comparison of genetic alterations among different molecular types

**Fig. 2:**
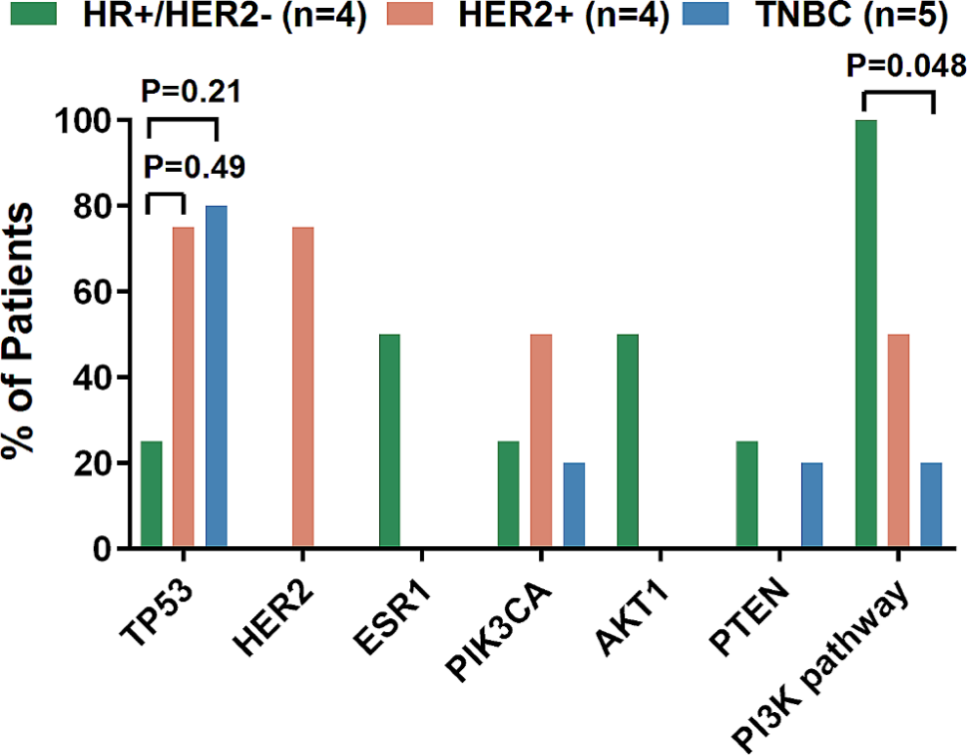
Molecular differences among three subgroups

Based on the HR and HER2 status from the pathological results of the primary lesion, patients were categorized into three subgroups including HR+/HER2- (4/13), HER2+ (4/13) and TNBC (5/13). To determine the genetic alteration characteristics of the subgroups, we compared the somatic mutations and related pathways using 9 primary tumors and 4 CM (primary tumor unavailable) (Fig. 2). The HER2 + group and TNBC group exhibited higher frequencies of *TP53* mutations compared with HR+/HER2- group, although the differences were not statistically significant (*P* = 0.49 and 0.21, respectively, Fig. 2). On the other hand, mutations in *ESR1* including p.D538G and p.L536P were observed only in HR+/HER2 + group. Furthermore, the frequency of alterations in PI3K pathway was significantly higher in HR+/HER2- group compared to TNBC (100% vs. 20%, *P* = 0.048).

Different colors represent HR+/HER2+, HER2 + and TNBC subgroups. The frequency of the 5 genes with the smallest P values and the only pathway with *P* < 0.05 among the three subgroups were presented in the figure. The P values that were discussed in our result were labeled in the figure. Other P values were all > 0.05, and were not labled in the figure.

### Potential mechanisms of cutaneous metastasis

**Fig. 3:**
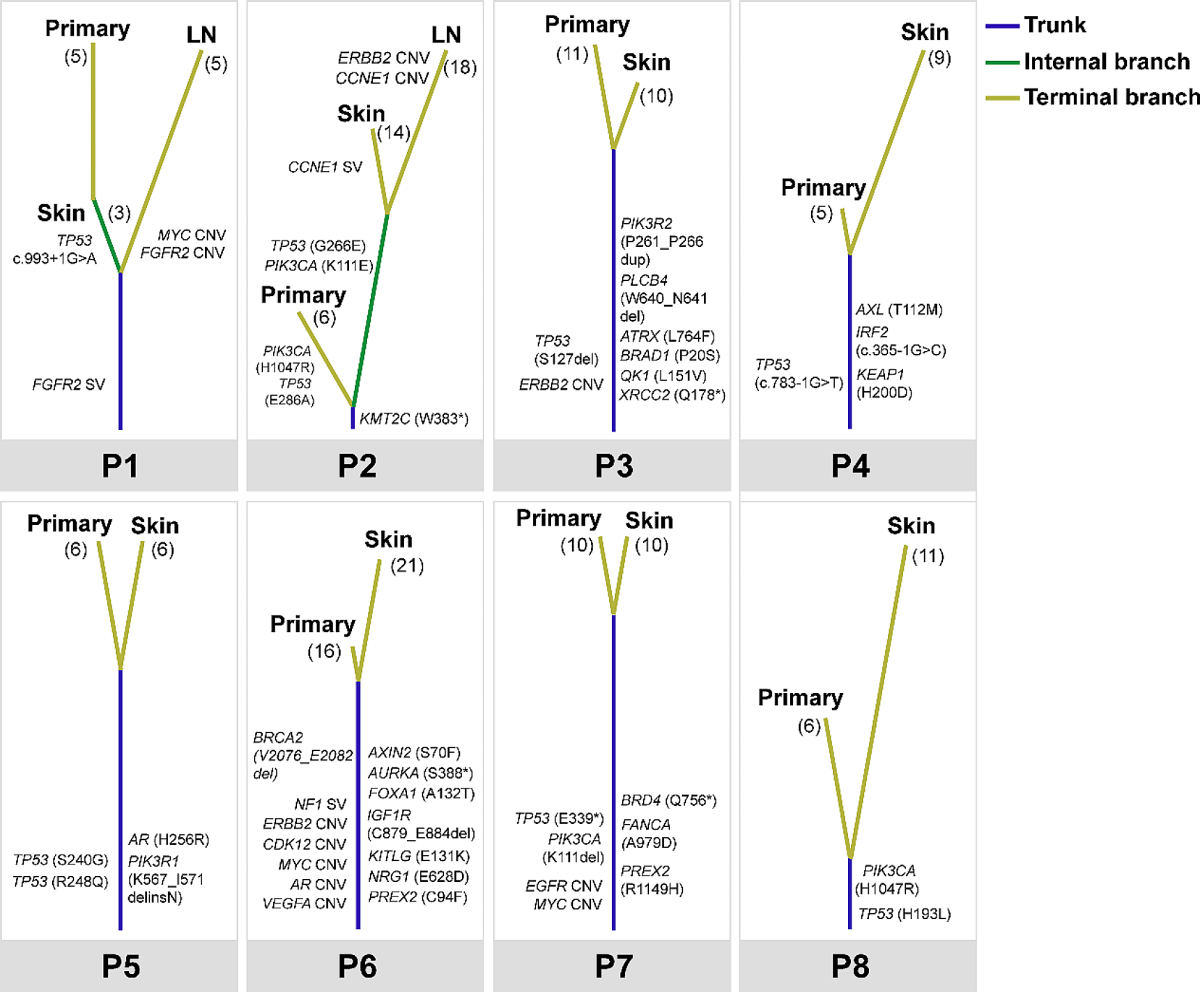
Phylogenetic trees of the 8 patients who had paired primary and metastasis (skin and LN)

We carefully compared the shared and unique mutations in paired primary and metastasis tumors (skin and LN) in the 8 patients (Fig. 3). All the shared variations in 2 or 3 samples of each patient were defined as trunk mutations, as labeled in Fig. 3. The specific variations were defined as branch mutations. *CBLB, BAK1, FLT1, ATRX, CREBBP, CHD8, PDK1, ALK, EZH2, MRE11A, SRC, ADGRB3, GATA3, XPA, PLCB4, DPYD, PTCH1, AXIN2, MET, ERBB4, FOXA1, NOTCH1, AMER1, ARID1A, ATR. EGFR, NSD1, RAD51C, FOXP1* mutations; *MYC, FGFR1/4, CDK12, TERT, AR* copy number gain and *STK11* copy number loss were found specifically in CMs. For these branch mutations, only potential driver genes associated with breast cancer were labeled in the figure. For all the 8 patients, at least one driver gene mutations were found in trunk mutations. However, only 2 patients had driver gene mutations in specific mutations: *TP53* mutation in CM of P1; *PIK3CA* and *TP53* mutations, *CCNE1* SV, and *ERBB2*, *CCNE1* CNV in primary tumor, CM and LN, respectively.

It showed the relationship of different primary tumors and metastasis (skin and LN) tumor from one patient. The length was proportional to the number of nonsynonymous somatic mutations. All mutations in trunk and potential driver mutations in branches were labeled on the phylogenetic tree. The length of the lines represents the relative magnification for each patient. The total number of nonsynonymous somatic mutations, CNV and SV of each sample were labeled in the brackets.

**Fig. 4:**
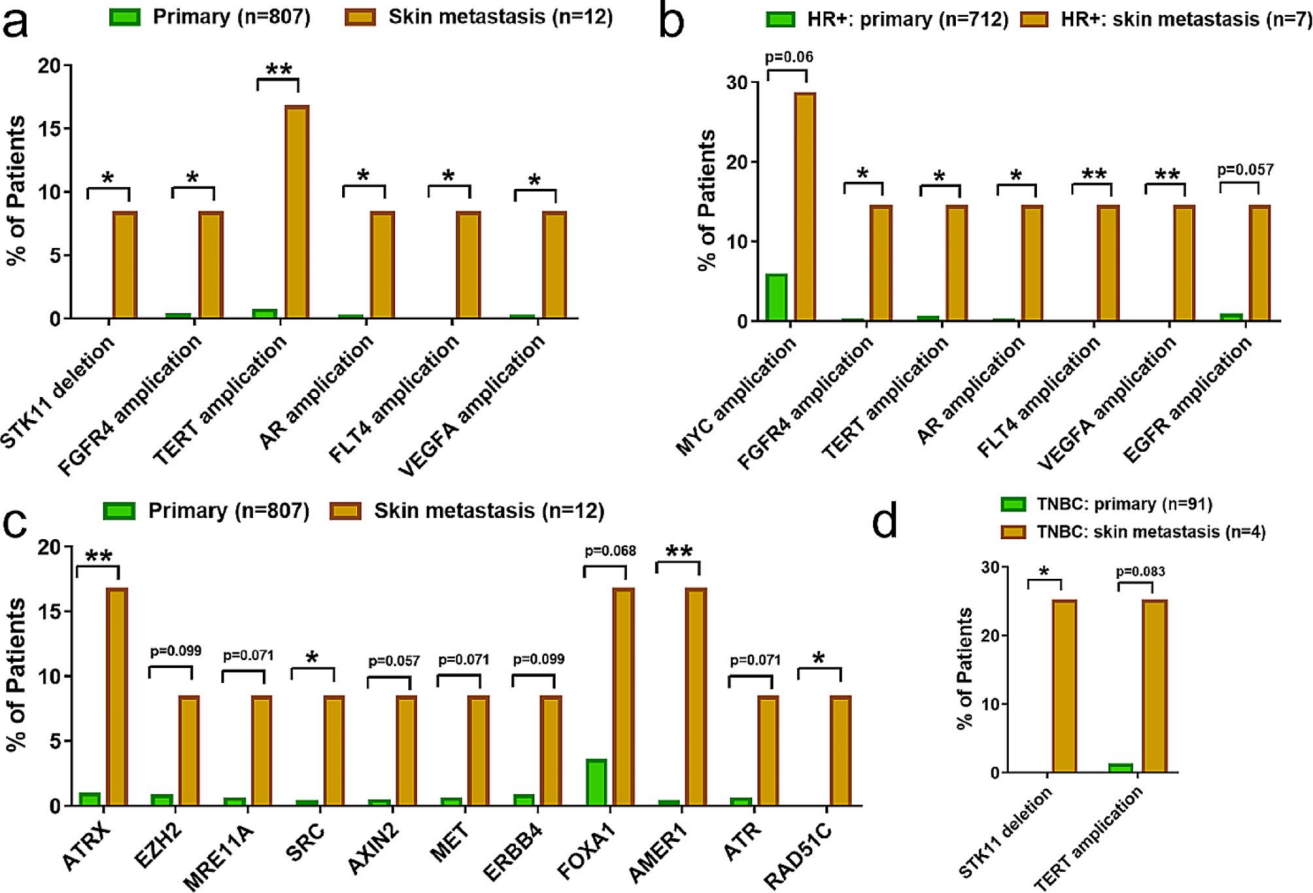
Molecular difference between primary and CM samples validated by TCGA dataset. Copy number alterations (**a**) and gene mutation (**c**) enriched in CMs, compared to primary breast tumors. Copy number alteration differences between primary and CM samples in HR+ (**b**) and TNBC (**d**) subgroups. The significance of gene enrichment in CM versus primary tumors was assessed using the long-rank test. P value < 0.05 was considered statistically significant. * and ** represent P value < 0.05 and < 0.01, respectively

In order to further evaluate the specific mutations in CM, we used an external dataset from a published study in which 807 primary breast tumors were analyzed [15]. We compared the genetic alterations between these primary tumors and 12 cm in our study. The clinical characteristics of this primary tumor cohort are shown in Table S3. Unlike our cohort, the HR+/HER2- subgroup accounted for the majority (81.8%) of these patients. Copy number loss in *STK11*, copy number gain in *FGFR4, TERT, AR, FLT4* and *VEGFA*, as well as mutations in *ATRX, SRC, AMER1* and *RAD51C* were significantly enriched in CM (all *P* < 0.05) (Fig. 4a, c). The majority (87.36%) of patients in primary tumor cohort were HR+, therefore we compared the altered genes in the HR + subgroup. Copy number loss in *STK11* and copy number gain in *FGFR4, TERT, AR, FLT4* and *VEGFA* remained enriched in CM within the HR + subgroup (all *P* < 0.05) (Fig. 4b). In TNBC subgroup, only the frequency of copy number loss in *STK11* was found significantly higher in CMs (Fig. 4d).

### The prognosis of breast cancer patients with CM

The patients with CM were reported to have a poor prognosis, regardless of the primary tumor site. We analyzed the survival status of the 13 patients by segment. T1 was defined as the time between diagnosis and the presentation of CM, and it was also defined as the disease free interval (DFI) of CM; T2 was defined as the time from the presentation of CM to death, and it was also defined as the overall survival (OS) The median time from diagnosis to the presentation of CM (T1, DFI) was 15 months (range: 0–94 months) and the median time from CM to death (T2, OS) was 13 months (range 1–78) (Fig. S2a, b). It was worth noting that the patient who had the longest T2 (78 months) had no other metastatic site other than the skin.

**Fig. 5:**
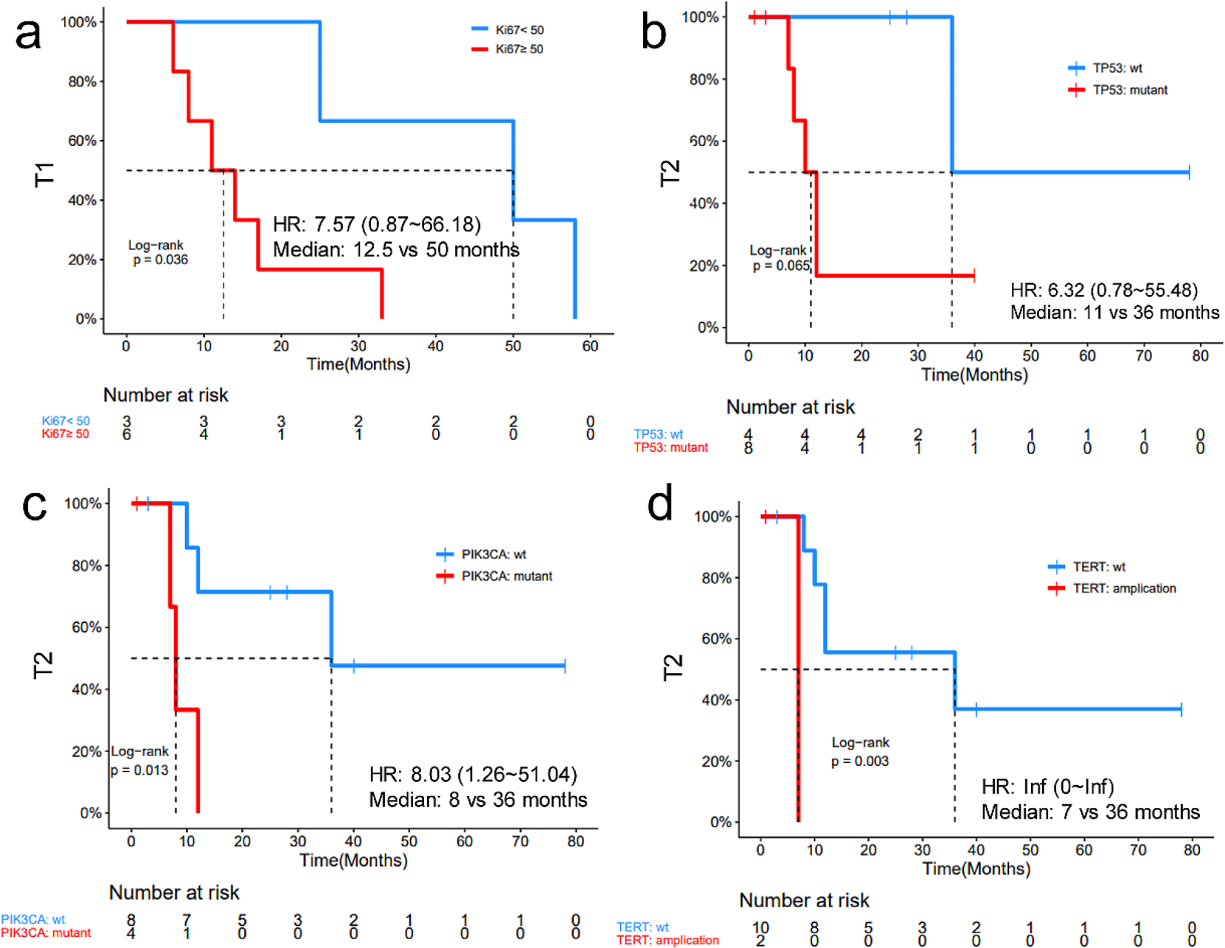
Clinical and molecular biomarkers of T1 and T2. (**a**) Association of high Ki67 expression level with the high risk of CM occurrence. Poorer survival (T2) was observed in patients harboring *TP53* mutation (**b**), *PIK3CA* mutation (**c**) and *TERT* amplification (**d**). The range of median T1/T2 were shown in the panels

The association of T1 and T2 with various clinical and molecular factors was evaluated. Ki67 high group (> 50%) showed significantly shorter T1 than the Ki67 low group (≤ 50%) (median 12.5 vs. 50.0 months, *P* = 0.036) (Fig. 5a). However, no significant correlation between T1 and molecular factors was observed. By contrast, *TP53* and *PIK3CA* mutations were associated with inferior T2 (median 11 vs. 36 months, *P* = 0.065; 8 vs. 36 months, *P* = 0.013, respectively) (Fig. 5b-c). *TERT* amplification group also exhibited a shorter T2 compared with *TERT* wide type (WT) group (median 7 vs. 36 months, *P* = 0.003) (Fig. 5d). These results suggested that *TP53, PIK3CA* mutant and *TERT* amplification might be molecular biomarkers for poor prognosis for patients with breast cancer and CM.

## Discussion

In this study, we aimed to explore the molecular alterations involved in the development of cutaneous metastasis in breast cancer. We studied the mutational landscape of 13 matched primary tumors with their lymph nodes or CM tissues using NGS. Overall, we observed a higher number of alterations in CM compared to primary tumors, with CM harboring more unique alterations. These findings are consistent with a previous studies, in which nearly half of CMs had additional alterations [4]. Similarly, another study reported a substantial overlap in alterations between matched primary tumor and metastases, and the number of alterations in metastases was higher than the corresponding primary tumors [13]. In our study, the most frequently altered genes were *TP53, PIK3CA, MYC* and *ERBB2*. *TP53* was more frequently detected in TNBC, while *PIK3CA* was more frequently found in HR + breast cancer patients, which were also consistent with the above two studies.

The common metastatic sites of breast cancer include the liver, brain, lymph nodes, lung, soft tissue, bone, ovary, and skin. Gene mutations in *TP53, KMT2C, RUNX1, AKT1, ESR1, XIRP2, PEAK1, PALB2, MYLK, EVC2* and *SLC2A4RG* have been reported to be associated with breast cancer metastasis, and their mutation frequency was significantly higher in metastases than in primary tumors. The copy number variations implicated in breast metastasis include *STK11* and *CDKN2A* copy number loss, as well as *PAQRB* and *PTK6* copy number gain [11]. In other studies comparing fewer than 10 pairs of primary and CM tumor, specific variations in CM included *ATR, BRCA1, SMAD4, CDH1, ARID1A, ERBB2, IDH1, PIK3R1, RB1*, and others, such as amplification of *FGFR1*/structural variant of *TP53*, indel of *RB1*/amplification of *TERT, JAK2, NF1, TP53, AKT1* and *ARID1A, PIK3CA, TP53*, and others [11, 12, 15]. Moelans et al. analyzed 22 cases with primary and cutaneous metastases from 55 primary BC samples and their corresponding distant metastases. They showed a higher frequency of CNVs in BC metastases compared with primary tumors. These genes were involved in various pathways, including the development of treatment resistance [16]. However, in our cohort, we only observed a difference in the frequency of the PI3K pathway between primary and CM tumors, possibly due to the small number of patients. By comparing the gene variation results of primary tumors with those published in the TCGA database, we also observed that the frequency of *STK11* loss, *FGFR4, TERT, AR, FLT4* and *VEGFA* gain, *ATRX, SRC, AMER1* and *RAD51C* mutations were significantly higher (*P* < 0.05) in CM, consistent with a previous study that associated these alterations with metastasis [11]. Additionally, other studies have identified 8 specific genes associated with skin metastasis: KRT14, KRT5, S100A7, SERPINB5, MMP3, IL20RB, SFN, TPSAB1 [17]. Summarizing the above researches, our results indicated that, apart from TERT amplification, no other alterations had been reported in the previous literature regarding CM. A larger cohort of breast cancer with CM is requiered to further validate these findings.

The new genetic variants found in metastases hold the potential to uncover noval targets for patients with skin metastases. Whether in HR + or triple negative breast cancer, the frequency of copy number loss in *STK11* was significantly higher in CMs. Serine/threonine kinase gene (*STK11*) functions as a tumor suppressor gene, and its mutation can lead to Peutz-Jeghers syndrome (PJS) [11]. The deletion of *STK11* may result in the activation of mTOR pathway [11], suggesting that mTOR inhibitors may potentially serve as therapeutic targets for this type of patients. *PIK3CA* was one of the most frequently altered genes across the three types of tissues. Patients with *PIK3CA* mutations should receive more attention as they may benefit from PI3K inhibitors [18]. Both our study and previous studies had found that the amplification of TERT is enriched in skin metastases. Telomerase reverse transcriptase (TERT) exerts a series of fundamental functions that are independent of its enzymatic cellular activity, including proliferation, inflammation, epithelia-mesenchymal transition (EMT), angiogenesis, DNA repair, and gene expression [19]. TERT amplification is associated with tumor metastasis and poor prognosis.

Patients with CM have very poor prognosis, which may be explained by their multi-organ metastases [4]. In this study, we found that the 13 patients were almost accompanied by other organ metastases besides CM. They had a short metastasis time (T1, median 15 months), and an even shorter T2, from the presentation of CM to death (median 13 months). Kong et, al reported that 56.8% of the patients with breast cancer had more than one visceral metastasis at the time of diagnosis of CM, with median survival time of 3 to 6 months. Minimal differences were observed in survival time between patients with single or multiple lesions, with the mortality rate exceeding 70% within the first year after diagnosis [20, 21]. Our study also found that 12 out of the 13 patients had metastases from other organs, except for one patient. Generally, patients with only lymph node and bone metastasis have a better prognosis. However, in our study, there were five patients with only bone and lymph node metastasis in addition to skin metastasis, and the median survival is only about one year. It may also indicate that the prognosis of patients with skin metastasis is worse, but the sample size is too small. Silvia et al [4] reported a median metastasis time of 22.8 months in 33 patients with BC and CM. This difference may arise from the different proportions of enrolled HER2 + patients, who may receive intensive treatment leading to longer remission periods. Furthermore, altered *TP53* and Ki67 have been reported as an independent prognostic factor. Indeed, a trend of poorer prognosis was observed in patients with *TP53* alterations in our study (*p* = 0.065). This could be due to the small sample size, which may not have resulted in significant differences. Patients with Ki67 high expression developed CM earlier in our study. Hepatocellular carcinoma (HCC) harboring *TERT* amplification has a poor prognosis [22], patients carrying *TERT* amplification in our study also exhibited a worse prognosis. Due to the small sample size and the lack of adjustment for other common prognostic parameters, the above conclusions are not yet definitive and require verification with larger sample sizes.

There are some limitations of our study. Firstly, the cohort size was relatively small, so the results should be interpreted with caution. Secondly, no patient in our study had Ki-67 levels less than 20%, which may introduce bias. Thirdly, due to the rarity of the CM sample and publications, we were unable to identify a suitable external cohort with NGS data to validate some of our findings.

Overall, our study compared the genomic features of primary breast cancer tissues with their corresponding CM tissues and discussed potential genes and pathways that may contribute to the skin metastasis of advanced breast cancers patients. Despite the limitations of our cohort, our findings may expand understanding of these specific patients and facilitate the decision of precise medication.

### Electronic supplementary material

Below is the link to the electronic supplementary material.

Supplementary Material 1

Supplementary Material 2

Supplementary Material 3

## Data Availability

No datasets were generated or analysed during the current study.
