# Supplementary figures and images for: Molecular alterations and prognosis of breast cancer with cutaneous metastasis
Source: Diagn Pathol. 2024 Jul 5;19:93. doi: 10.1186/s13000-024-01509-x (PMC11225245; doi:10.1186/s13000-024-01509-x)

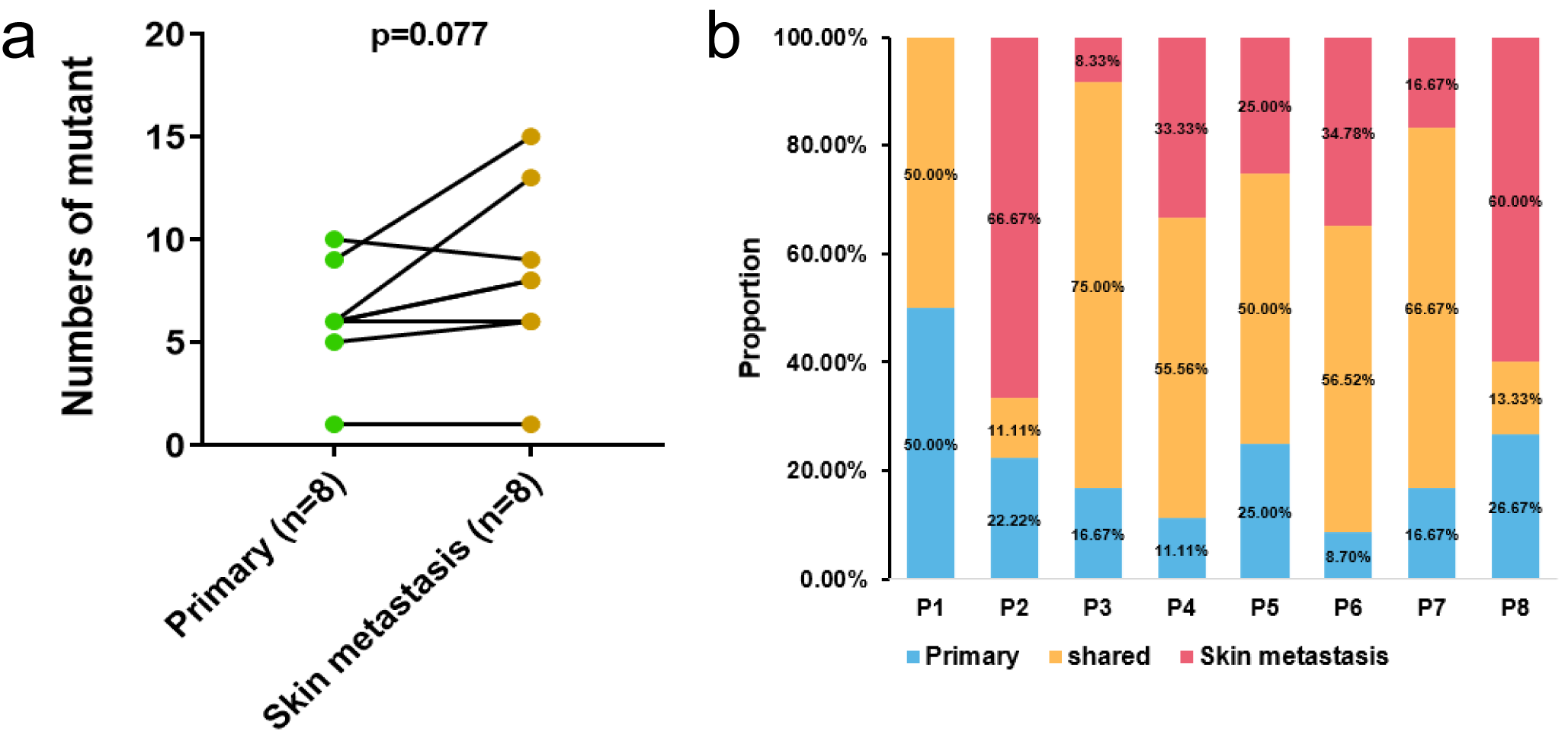

Supplement: Supplementary file 1 — Supplementary Material 1 [file 13000_2024_1509_MOESM1_ESM.tif]

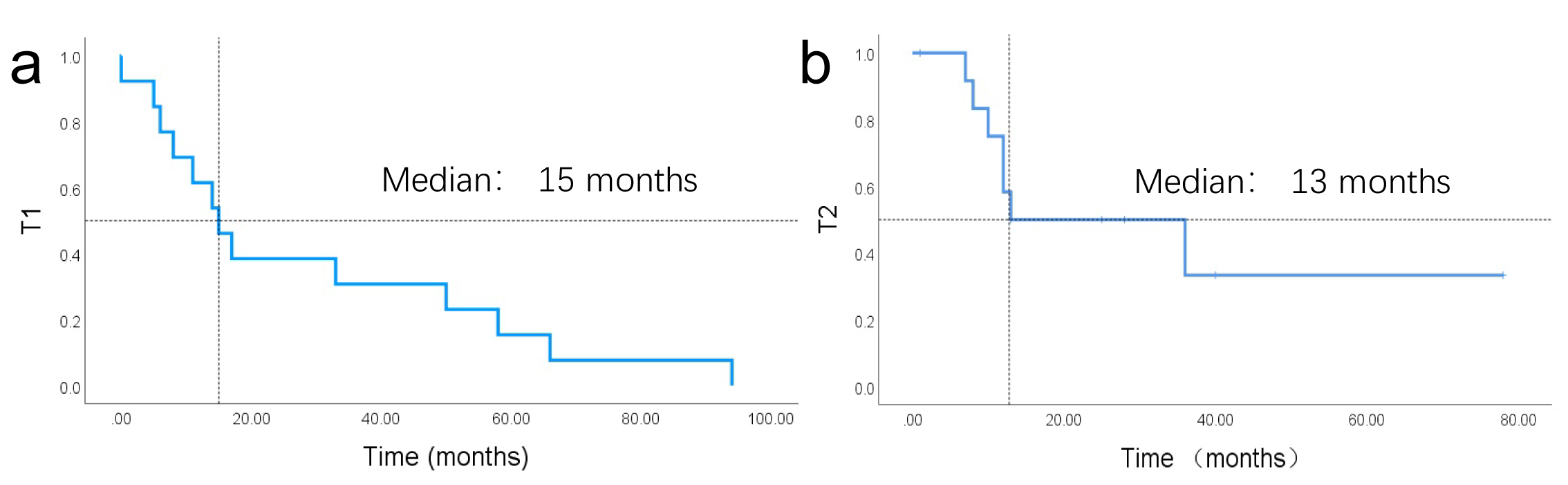

Supplement: Supplementary file 3 — Supplementary Material 3 [file 13000_2024_1509_MOESM3_ESM.tif]
